# Reverse cholesterol transport: current assay methods, alterations with disease and response to therapeutic intervention

**DOI:** 10.3389/fcvm.2025.1608384

**Published:** 2025-07-10

**Authors:** Martin P. Playford, Edward B. Neufeld, Rafael Zubirán, Alan T. Remaley

**Affiliations:** ^1^ Section of Inflammation and Cardiometabolic Diseases, National Heart, Lung and Blood Institute, NIH, Bethesda, MD, United States; ^2^ Lipoprotein Metabolism Laboratory, National Heart, Lung, and Blood Institute (NHLBI), NIH, Bethesda, MD, United States

**Keywords:** cholesterol, ABCA1, ApoA-1, inflammation, reverse cholesterol transport (RCT)

## Abstract

The removal of excess cholesterol from the body by High-density lipoprotein (HDL) in a process termed reverse cholesterol transport (RCT) has long been proposed to play a critical role in reduction of the lipid burden in arterial wall atherosclerotic lesions. While HDL-cholesterol levels are associated with decreased cardiovascular risk and considered to be “good-cholesterol”, clinical studies using HDL-raising therapies to potentially enhance RCT have consistently produced disappointing results. In this mini review we evaluate the effects of human disease on RCT along with the changes in this process upon various therapeutic interventions. Despite the importance of assay standardization, the major method for monitoring RCT has relied upon the cholesterol efflux capacity (CEC) assay, a highly-difficult and tedious cell culture assay, which is low-throughput and only suitable for research studies. Hence, we also briefly review several new methods to measure RCT both *in vitro* and *in vivo,* along with new cell-free alternative RCT assays, which have the potential to be developed into routine automated diagnostic assay. The benefits of HDL may yet be revealed by the use of these new high-throughput RCT assays perhaps as a screening tool for novel RCT boosting agents or as new biomarkers for cardiovascular disease risk.

## 1 Introduction

High-density lipoprotein (HDL) is a spherical complex made up of a heterogenous array of proteins and lipids, as well as small RNA molecules (1–4). Throughout its lifecycle, HDL undergoes continuous recruitment, assembly, delivery and turnover of its various protein and lipid components (5). With such a diversity of cargo, HDL is believed to have functions in numerous biological processes, such as in oxidation, inflammation and diabetes (3). HDL also removes cholesterol deposited in the arterial wall by LDL by both active and passive efflux processes that are commonly measured as its cholesterol efflux capacity (CEC). Subsequently, cholesterol on HDL is either directly taken up by the liver or is indirectly removed by the liver after being transferred to LDL by cholesteryl-ester transfer protein (CETP) (6). Ultimately, cholesterol delivered to the liver is either reutilized or is excreted into the intestine in the bile either as free cholesterol or as bile salt after conversion. The final subsequent removal of cholesterol from the body completes a process called Reverse Cholesterol Transport (RCT) by John Glomset in the late 1960s (3, 7). Given that many of the individual steps in RCT are potentially atheroprotective, and supported by numerous epidemiologic studies demonstrating an inverse-relationship between HDL-C levels and the risk of developing coronary heart disease, cholesterol on HDL became commonly referred to as ‘good’ cholesterol (8, 9). Based on this evidence, numerous HDL level-boosting agents were tested therapeutically as an approach to reduce cardiovascular disease (CVD) [summarized in (5, 9)].

Despite the seeming biologic plausibility of the atheroprotective role of RCT, none of HDL- based therapies tested to date have translated to human benefit (5, 10). The most recent clinical trial (AEGIS-II) infused CSL112 (reconstituted HDL) or placebo control in a total of 18,219 patients with acute myocardial infarction, coronary artery disease and other cardiovascular risk factors with the 90-day composite end point being either myocardial infarction, stroke or death (11). CSL112 was formulated with ApoA-1 purified from plasma and combined with phospholipid to form a reconstituted form of HDL, which both *in vitro* and *in vivo* studies was demonstrated to increase RCT (11, 12). Unfortunately, this clinical trial found no significant differences in the primary outcome measures for cardiovascular events between the CSL112 and the placebo treated groups (11). While these accumulating trial failures have diminished enthusiasm for HDL-raising agents as a CVD therapeutic target, a number of possible explanations have been proposed for these negative findings, which should be addressed before the RCT hypothesis is abandoned (13, 14).

One important limitation of the AEGIS-II study is that patients lacked both baseline and follow-up measurements of CEC (11, 13). Though this information would have been valuable for this study, the lack of suitable technology for performing high-throughput large-scale measurements of CEC made this unfeasible. However, recent reports have described new approaches for performing CEC assays, which may point to a future where CEC measurements are a routine diagnostic tool in the clinical laboratory. This review provides a brief history on the variety of methods used to assess RCT in the laboratory (Section 1) and the new cell-free approaches (Section 4). We also provide an overview of how HDL function is affected by many human diseases and disorders (Section 2), in response to therapeutic intervention (Section 3) and the HDL proteome (Section 5).

## 2 A brief history of RCT measurement in the laboratory

RCT was first studied in human and animal *in vivo* studies via compartmental modeling of the distribution of *i.v.* injected radiocholesterol in plasma lipoproteins, red blood cells and the liver (15). Subsequently models involving cholesterol RCT measurement of 3H-cholesterol or cholesterol mass changes in macrophages implanted *i.*p*.* in mice were developed and provided evidence for HDL-mediated RCT from macrophages *in vivo* (16, 17).

**Figure 1:**
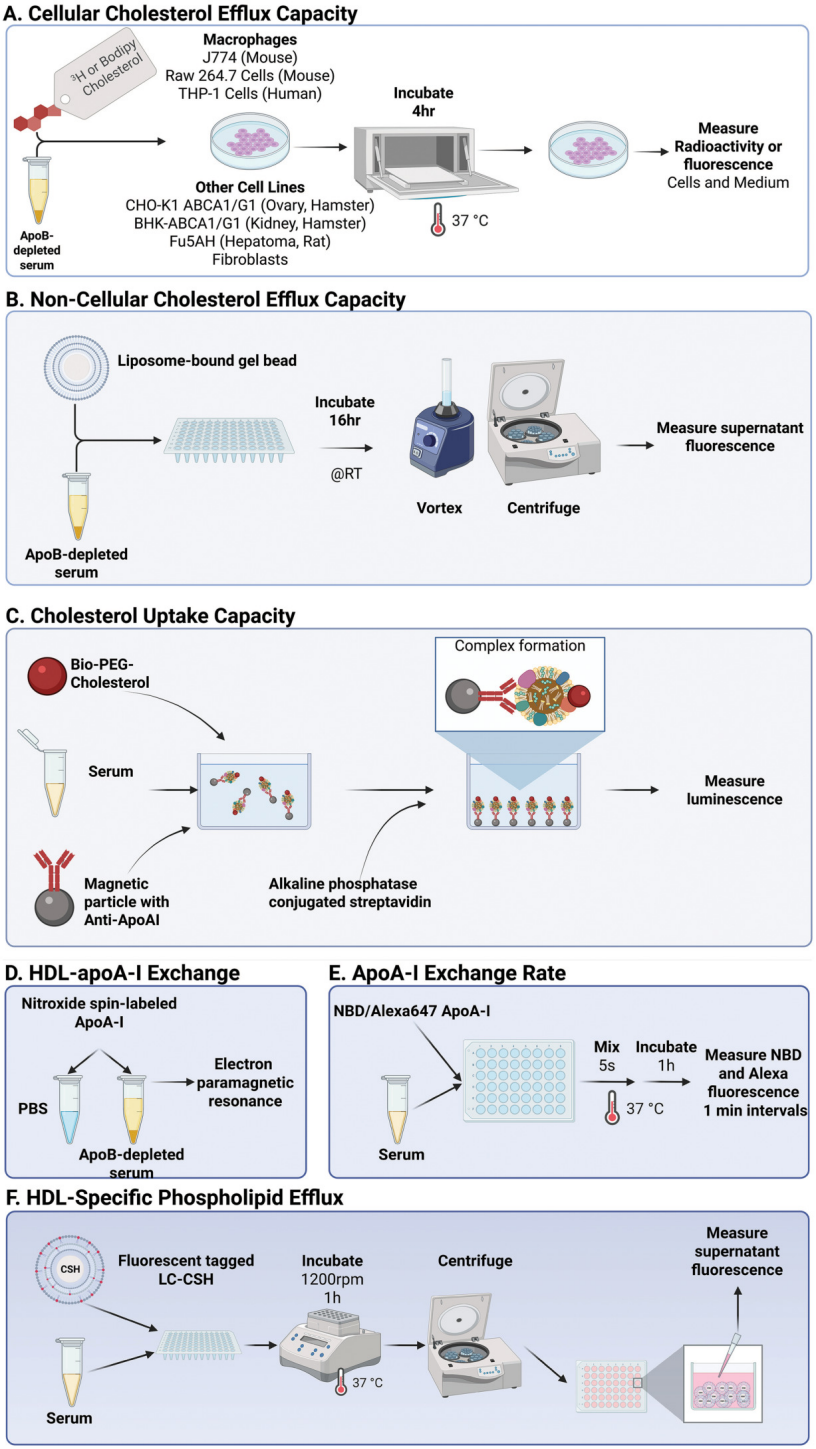
Cholesterol Efflux based *in vitro* RCT surrogate assays **(A–C)** and ApoA-I exchange-mediated *in vitro* RCT surrogate assays **(D–F)**. Schematized representations of surrogate assays for the initial step(s) in RCT. **(A)** Cellular Cholesterol Efflux Capacity, the “gold standard” *in vitro* method to measure RCT requires days of cell culture, radiocholesterol, apoB- depleted serum and is not amenable to translation to a clinical diagnostic assay. Cell-free cholesterol efflux assays. **(B)** The “Non-Cellular” Cholesterol Efflux Capacity assay replaces cells with immobilized liposome-bound gel beads (ILG) as a donor of fluorescent cholesterol to apoB- depleted plasma and requires overnight incubation. **(C)** The Cholesterol Uptake Capacity is an automated multi-step assay that requires apoA-I-mediated capture of Biotin-PEG3-cholesterol- labeled serum HDL onto magnetic beads and then detection of luminescence of biotin-bound alkaline phosphatase-conjugated streptavidin. **(D)** Exchange of exogenous apoA-I to plasma HDL: The HDL-apoA-I Exchange assay measures electron paramagnetic resonance of exogenous nitroxide spin-labeled apoA-I that exchanges onto plasma HDL. **(E)** The ApoA1 Exchange Rate assay measures the kinetics of dual fluorescent emission of plasma HDL- associated exogenous apoA-I labeled with NBD (cholesterol-sensitive) and Alexa647 (non- cholesterol-sensitive reference). **(F)** The HDL-specific phospholipid efflux assay monitors solubilization by endogenous apoA-I and other exchangeable HDL apolipoproteins in whole plasma, of a non-transferable fluorescent phospholipid from phospholipid/cholesterol coated nanoparticles. Figure created in BioRender. Zubiran, R. (2025) https://BioRender.com/k10f072.

It has been almost fifteen years since the first cell-based assays were developed to quantify the initial step of RCT (18, 19) (Figure 1), namely CEC. These early CEC assays of HDL function typically utilize mouse macrophage J774, Raw 264.7 cells or human Thp-1 cells, which are either radiolabeled with 3H- or fluorescently-labeled with BODIPY fluorophore (dipyrrometheneboron difluoride) and then incubated with apoB-depleted sera for a short time period, typically 4 h (Figure 1A). Additionally, specific CEC pathways related to ABCA1, ABCG1 and SRB1 may be revealed by CEC assays in Baby Hamster Kidney (BHK), Chinese Hamster Ovary (CHO) or HeLa cell lines with stable expression of these proteins that also affect the efflux of lipids from cells (20). An index of CEC is then calculated either by using a ratio to a pool of healthy control subjects or a percentage apoB-depleted serum of a cellular total of cholesterol label (21, 22).

Detailed methodological aspects of the cell-based assays are thoroughly discussed elsewhere (23, 24). It should be noted that these assays are technically challenging. They are not only affected by cell-density but also cell adherence, which given the semi-adherent nature of J774, Raw 264.7 and Thp-1 cells make experimental reproducibility a problem (24). The relatively large assay-to-assay variability may best be mitigated by comparison of a control pool of healthy subjects with each test sample. Another alternative is to measure CEC in radiolabeled cells to purified HDL, isolated by either ultracentrifugation or size-exclusion chromatography (25, 26), which is perhaps a more accurate measure of CEC but is time-consuming.

## 3 Human conditions and their effect on RCT

Numerous reports have shown that systemic and vascular inflammatory states can impair RCT via perturbed CEC function, which consequently promotes plaque progression (27, 28). The inter-relationship between RCT and both inflammatory and non-inflammatory states is highlighted by a detailed evaluation of the effect of human diseases and disorders on RCT (Table 1).

**Table 1:** Reverse cholesterol transport: alterations with human disorders.

Condition/ disease	Human condition/ disease type	Reference	PMID	year	Cells for efflux	Cholesterol label	Numbers of subjects	Change from control
AD	Alzheimer's disease	Marchi C et al.	31167810	2019	J774 and CHO-K1-ABCG1	3H	AD 37, control 39	CSF from AD patients had lower ABCA1 CEC 73%
AS	Ankylosing spondylitis	Gkolfinopoulou C et al.	26233507	2015	J774	3H	35 AS and 35 controls	AS patients have decreased ability to efflux cholesterol
BD	Schizophrenia and bipolar disorder	Hussein O et al.	26253619	2015	J774	3H	19 test, 10 control	Test subjects down 20%
CHD	Coronary heart disease	Saleheen D et al.	26025389	2015	J774	3H	1745 disease, 1749 control	CEC inversely correlated with T2D, positively correlated with alcohol consumption
CKD	Chronic kidney disease	Chindhy S et al.	30072004	2018	J774	BODIPY	210 baseline CKD vs. 2595 no CKD	No change
CKD	Chronic kidney disease	Holzer M et al.	21804091	2011	Raw 264.7	3H	27 hemodialysis (HD) patients, 19 controls	HD patients have significantly lower CEC to pure HDL
COVID	COVID-19	Mietus-Snyder M et al.	36312284	2022	J774	3H	30 control, 38 mild disease, 33 severe disease, 31 MIS-C	CEC capacity decreased in proportion to disease severity. MIS-C decreased 31% from control
COVID	COVID-19	Stadler JT et al.	36290581	2022	J774	3H	47 COVID-19, 32 control non-COVID pneumonia	CEC lower in COVID-19 patients. CEC inversely associated with risk of mortality
Crohns	Crohns disease	Franssen R et al.	22611487	2012	Thp-1 and fibroblasts	3H	10 test, 5 control	No change
CVD	Atherosclerosis	Khera AV et al.	21226578	2011	J774	3H	442 CAD, 203 healthy	Control 0.9, test 0.82. 10% decrease
CVD	Diagnosed CVD or one CV factor	Noflatscher M et al.	37509557	2023	J774	3H	176 subjects with risk factor or pre-existing CVD	Inverse correlation between CEC and high peripheral plaque volume
CVD	Acute coronary syndrome	Hafiane A et al.	24210679	2014	J774	3H	20 ACS, 9 healthy	Control 25.9%, ACS baseline 16.9
CVD	STEMI	Annema W et al.	27919348	2016	Thp-1	3H	41 non-STEMI, 37 STEMI, 33 non-MI	11% decrease in STEMI compared to non-STEMI or NON-MI
CVD	Acute heart failure	Pammer A et al.	39244794	2024	J774	3H	315 AHF patients (74 deceased, 241 alive)	CEC significantly reduced in deceased AHF patients
CVID	Common variable immunodeficiency	Macpherson ME et al.	31263122	2019	Thp-1	14C	102 CVID, 28 healthy	Healthy CEC 15%, CVID CEC 14.1%
FH	Familial hypercholesterolemia	Bellanger N et al.	21527752	2011	Thp-1	3H	12 test 12 control	27% defect in efflux to HDL-2 in FH patients. No change with HDL-3
FH	Familial hypercholesterolemia	Ogura M et al.	26543100	2016	J774	3H	227 FH, 76 with additional CVD	12% CEC defect compared to control pool, CEC inversely associated with CVD
FH	Familial hypercholesterolemia	Sato M et al.	39048165	2024	HDL-SPE	PE	30 FH, 60 age- and sex- matched controls	HDL-SPE in control 1.12, FH patients 0.9
GS	Gilbert's syndrome (high bilirubin)	Wang D et al.	28455345	2017	Thp1	3H	60 test, 60 control	FH down 7.2%
H.pylori	Helicobacter. pylori infection	Fallah S et al.	33250430	2021	J774	3H	44 test, 43 control	HP 10.06% down when adjusted for age, sex and BMI
HF	Heart failure	Potocnjak I et al.	27304214	2016	J774	3H	152 heart failure patients	Low CEC inversely associated with hospital mortality (odds ratio = 0.78)
HIV	HIV	Munger AM et al.	25416403	2015	J774	3H	118 test	Test 0.975, pool 1
IBD	Inflammatory bowel disease	Piquer BR et al.	16784973	2006	Fu5AH	3H	20 test (5 quiescent, 15 active), 20 control	Active IBD down 11.53%, quiescent down 13.64%
Met/Syn	Metabolic syndrome	Annema W et al.	27270665	2016	Thp-1	3H	297 Met S, 255 no Met S	1.29/1.38 in Met S. down 6.5%. Independent of plasma HDL-C
Met/Syn	Metabolic syndrome	Lucero D et al.	26232163	2015	BHK-ABCA1 or ABCG1	3H	35 Met S, 15 healthy control	Increase in ABCA1-mediated CEC in Met S patients (10.4%) vs. control (8.7%)
None	No history of CVD	Rohatgi A et al.	25404125	2014	J774	BODIPY	2924	67% decrease in CV risk for highest quartile from lowest. After adjustment
None	No history of CVD	Hunter WG et al.	37615111	2023	J774	BODIPY	3543	After adjustment, CEC inversely related to betaine and succinate. Positive with threonine and dimethylamine
NAFLD	Non-alcoholic fatty liver disease	Fadaei R et al.	30076407	2018	J774 and Thp-1	3H	55 NAFLD, 30 control	0.89 to 1.03 J774, 1.09–1.16 Thp1
OSA	Obstructive sleep apnea	Fadaei R et al.	36344946	2022	J774	3H	69 test, 23 control	OSA down 14.6%
PCOS	Polycystic ovarian syndrome	Roe A et al.	24512495	2014	J774	3H	124 PCOS, 67 control	Test 0.98, control 1.05
PSO	Psoriasis	Mehta NN et al.	22858285	2012	J774	3H	122 PSO, 134 healthy	Control 0.98, PSO 0.83.
PSO	Psoriasis	Holzer M et al.	23985995	2014	Raw 264.7	3H	15 PSO and 15 healthy	PSO down 20%
RA	Rheumatoid arthritis	Ronda N et al.	23562986	2014	J774 and CHO-K1-ABCG1	3H	30 control, 30 RA	RA down 15% to ABCG1,
RA	Rheumatoid arthritis	Vivekanadan-Giri A et al.	23313808	2013	J774	3H	20 control, 38 RA	RA down 21%
SLE	SLE	Ronda N et al.	23562986	2014	J774 and CHO-K1-ABCG1	3H	30 control, 30 SLE	SLE down 40% to ABCG1, down 24% to ABCA1
SLE	SLE	Sanchez-Perez H et al.	32065639	2020	J774	BODIPY	195 SLE, 223 controls	SLE 8.1%, control 16.9%
SSc	Systemic sclerosis	Ferraz-Amaro et al.	33622410	2021	J774	BODIPY	73 test, 115 controls	SSc 8%, control 17
Stroke	Ischemic stroke	Papagiannis A et al.	36657609	2023	J774	3H	102 severe stroke, 97 mild stroke 97	Lower CEC in patients with severe stroke than mild stroke
T1D	Type 1 diabetes mellitus	Majunatha S et al.	27506748	2016	J774	3H	15 test (with good glycemic control), 15 control	T1D down 6.1%
T1D	Type 1 diabetes mellitus	Gourgari E et al.	30567548	2018	J774	3H	78 test, 59 control	T1D down 6.7%
T2D	Type 2 diabetes mellitus	Apro J et al.	27034474	2016	Thp-1	3H	35 test 35 control	10% decrease in T2D
T2D	Type 2 diabetes mellitus	Denimal D et al.	35647758	2022	Thp-1	BODIPY	20 test, 25 control	No change

In brief, nearly 40 published articles, covering almost 30 different human conditions, have shown that HDL can become dysfunctional leading to decreased CEC. In the following few paragraphs, we highlight the several examples of this by describing HDL function in Inflammatory diseases, Atherosclerotic Cardiovascular Disease, Familial Hypercholesterolemia, Diabetes and Chronic Kidney Disease.

### 3.1 Chronic systemic inflammation on RCT/CEC

Chronic systemic inflammation plays a key role in the pathogenesis of a wide variety of human diseases, including diabetes (T1D and T2D), psoriasis, systemic lupus erythematosus (SLE), inflammatory bowel disease (IBD), fatty liver disease and several arthritic conditions. Low-grade systemic inflammation is also observed in patients infected with HIV, polycystic ovarian syndrome, schizophrenia and even in sleep apnea. Table 1 highlights the human studies that have measured RCT in these different inflammatory conditions.

Despite the heterogenous options to measure RCT/ CEC *in vitro*, a common theme across all these studies is the almost universal reduction of RCT/ CEC under all inflammatory conditions (Table 1) (29–40). For example, in a 2012 study using J774 cells loaded with 3H-cholesterol and apoB- depleted serum, CEC was reduced 15.3% in 122 psoriasis patients compared with 134 non- psoriatic controls (21). A similar finding was reported in a later study, where CEC was impaired 20% in 3H-loaded Raw 264.7 cells to purified HDL from 15 patients with psoriasis, compared to HDL from 15 age and sex matched healthy controls (25).

One inflammatory disease for which the effect on RCT remain inconclusive are in the bowel, but the studies are small and the CEC assays are performed differently. While Ripolles-Piquer *et al*. found a 11.5% decrease in CEC in 15 active IBD subjects using 3H-loaded rat hepatoma Fu5AH cells, Franssen *et al*. failed to note any difference in CEC between 10 Crohns patients and 5 controls when using Thp-1 cells as lipid-laden host cell (41, 42).

### 3.2 Atherosclerotic cardiovascular disease (ASCVD)

The shift in HDL research from just measuring HDL-C to assessing its functionality has gained interest as a potential tool for ASCVD risk prediction, as well as in providing guidance in the development of new HDL target therapies. Meta-analyses of CEC trials have shown consistently the inverse association between different cardiovascular outcomes, such as Coronary Artery Disease (CAD), unstable angina, non fatal MI, stenosis of major coronary vessels, coronary revascularization, stroke, cardiovascular death and all cause mortality (43, 44). The first report to establish the inverse relationship between CEC and CAD was done in a study by Khera et al. where (ATP-binding cassette transporter-dependent cholesterol efflux capacity (ABCA1-CEC) was measured in 203 healthy volunteers studied for subclinical atherosclerosis (assessed by carotid intima-media thickness) and 793 patients who underwent cardiac catheterization (442 case and 351 controls). In both cohorts, it was shown that CEC was a strong inverse predictor of CVD status; per 1-Standard Deviation (SD) increase of CEC there was a 30% less risk of CAD (45). Despite the reduction in levels of both HDL-C and ApoA-I between healthy volunteers and patients with CAD, adjustment for either factor caused the association between CEC and CAD to be attenuated yet remained significant (45). These findings were later replicated by Rohtagi et al. in 2,924 adults without CVD of the Dallas Heart Study where the highest quartile of ABCA1-CEC was associated with 67% less risk for developing ASCVD events (46). Later studies by Mody et al. showed that adding HDL-CEC to common CVD risk factors improved the net classification index for the prediction of ASCVD (47). In 2014, Saleheen et al. confirmed these findings in a nested case-control sample from the prospective EPIC-Norfolk study (48). This study showed how that the relation between ABCA1-CEC and incident coronary heart disease events were independent of multiple cardiovascular risk factors and remained significant after adjustment for HDL-C and ApoA-I (48). Similar findings have been shown in several clinical scenarios, such as acute coronary syndromes (49, 50), non fatal Myocardial Infarction (MI) (51), and stenosis of major coronary vessels (52), and coronary revascularization (53).

Despite strong associations at baseline, some longitudinal studies on CEC have provided contradictory findings (54, 55). For example, in a *posthoc* analysis on the Justification for the Use of Statins in Prevention: an Intervention Trial Evaluating Rosuvastatin (JUPITER) trial, CEC at baseline was not associated with incident CV events (56), and treatment group with rosuvastatin for 12 months did not change CEC while increasing HDL-C and Apolipoprotein A1 (ApoA-I), 7.7% and 4.3% respectively. Interestingly this study also revealed that those on statins HDL-CEC was inversely associated with incident ASCVD (56).

However, in this trial, an inverse relationship between inflammatory biomarkers and CEC was observed. These contradictory findings suggest that relationship between CEC and CVD could be population-specific and further influenced by multiple factors, such as HDL size, lipid free ApoA-1 in the Apo-B depleted serum, and the metabolomic profile of patients. The latter idea was recently studied by Hunter et al. in the MESA (Multi-Ethnic Study of Atherosclerosis) where using NMR metabolomic and lipidomic profile, they identified association of subclasses of VLDL and HDL, as well as their constituent ApoA-1, ApoA-2, phospholipid, and cholesterol components with CEC. Race, however, was found to be the most powerful predictor of CEC (57).

A recent metanalysis have concluded that higher CEC is associated with a lower risk for ASCVD. This study showed that the highest levels of CEC were associated with a 34% lower risk of ASCVD (RR = 0.66; 95% CI, 0.55–0.80; *P* < 0.0001), which remained significant after adjustment for other common CVD risk factors and HDL-C (RR = 0.79; 95% CI, 0.65–0.97; *P* = 0.02). This study also concluded that for each standard deviation increase in CEC there was a 20% lower risk for ASCVD (44).

It is important to note that the design of the above studies, follow-ups and definitions of cardiovascular events are somewhat heterogeneous. There are also several methodological limitations as some studies use THP-1 cells, whereas most others use J774 (mouse) as the host cholesterol-laden macrophages. In addition, a few studies label cholesterol with fluorescent sterols (BODIPY) as an alternative to the more frequent use of radioisotope labeling (3H or 14C).

### 3.3 Familial hypercholesterolemia

Familial hypercholesterolemia (FH), an autosomal dominant genetic disorder caused by mutations in Low-Density Lipoprotein Receptor (LDLR), ApoB or Proprotein Convertase Subtilisin/kexin type 9 (PCSK9), is characterized by life-long exposure of very high levels of LDL-C and early cardiovascular disease. Although there are no HDL-related genes affected in FH, many studies have shown quantitative and qualitative changes in HDL, leading to a change in its function (58). For example, it has been reported that individuals with Heterozygous FH (HeFH) have an independent and inverse association of ASCVD and ABCA1-CEC (59). Another study showed that compared to their non-affected siblings, individuals with FH without CVD had 16% ± 22% higher efflux and those with FH and CVD had 7% ± 8% lower (59). This effect could be partly explained by the changes in the HDL proteome in asymptomatic patients and could play a role in the development of early CVD in these patients. Using the HDL-specific phospholipid efflux (HDL-SPE) assay, a recent report demonstrated that FH patients (*n* = 30) had a near 20% efflux decrease compared to age- and sex- matched controls (60). Levels of HDL-C were similar between both cohorts (60).

### 3.4 Diabetes

HDL abnormalities in diabetes have been the subject of several recent reviews (61). Glycation of HDL in diabetes increases plasma clearance and the loss of the protein components (62, 63). Consistently, most studies have shown that compared to healthy subjects, CEC in patients with Type 2 diabetes (T2D) is reduced (48, 64). A study of a cohort of 640 patients with T2D matched to controls by HDL-C showed that there was a reduction in ABCA1-CEC (65). This was later supported by another study where reduced ABCA1-CEC of small HDL was likely due to the loss of the protein SERPINA1 (63). Other studies have speculated that the shift to smaller HDL particles in diabetic patients could be the reason for the lack of significant differences or even enhanced CEC in different studies of diabetics (66, 67).

Interestingly, patients with Type 1 Diabetes (T1D), as opposed to T2D patients, typically have higher levels of HDL-C, although their HDL is generally not considered to be as atheroprotective. Consistent with this, some studies have shown that compared to healthy subjects, those with T1D had smaller HDL size and lower CEC (40). However, glycemic control in patients with T1D was not associated with improvement on CEC. The effect of statin therapy on CEC is inconsistent. Some reports have shown pitavastatin enhanced CEC, whereas atorvastatin had no effect on CEC (68, 69).

It should be noted that there are inconsistent findings in the reporting of RCT/CEC for T2D. Using 3H-loaded Thp-1 cells, Apro et al. reported a 10% decrease in CEC in 35 T2D subjects compared to 35 controls, whereas Denimal *et al*. using similar cells, but labeled with BODIPY, failed to note a change in CEC between 20 subjects and 25 control (38, 39). Taken together, the effect of T2D on CEC studies remains inconclusive and would benefit from further studies with larger numbers of subjects and performed either in 3H-cholesterol labeled J774 cells or using a cell-free assay, as described below.

### 3.5 Chronic kidney disease and kidney transplant

Chronic kidney disease (CKD) is considered one of the most common risk factors for CVD. Despite being most commonly caused by hypertension and diabetes, CKD is recognized as an independent risk factor for CVD (70). Within the different changes that happen during CKD, dyslipidemia is one of the most frequent. Low HDL-C levels are present in 42% of patients with CKD. Structural modifications in HDL that occur in CKD include enrichment in Serum Amyloid A1 (SAA1) and ApoC-III and the depletion in apoA-I, apoA-II, and phospholipids, all of which could impede HDL function (70). It has been consistently shown that CKD patients with albuminuria or low estimated.

glomerular filtration rate have impaired ABCA1-CEC (71). It has been even shown in the Dallas heart study that in those without CVD a higher CEC at baseline may predict an increased CVD risk in CKD patients, and CEC was found to be negatively associated with incident CVD in subjects without CKD (72). In more advanced CKD, such as in patients on dialysis, changes in the PL and TG content of HDL are shown to correlate with impairment in CEC. In particular the depletion of PL was found to be associated with reduced content of ApoA-I and ApoA-II, due to displacement by SAA, leading to impaired cholesterol efflux capability. Despite the inverse relationship of CEC with ASCVD events in other conditions, a recent metanalysis did not observe this relationship in CKD patients (RR = 1.08; 95% CI, 0.86–1.38; *P* = 0.50) (44). Interestingly, other studies have found that CEC did not independently predict risk for cardiovascular disease and all-cause mortality after kidney transplantation. Improved CEC was, however, associated with protection against graft failure (71).

### 3.6 RCT/ CEC in cancer and other disorders

Alterations in cholesterol transport have also been shown to facilitate the pathogenesis of cancer, age-related macular degeneration and Fragile X (73–75). For example, a recent report has demonstrated that impaired RCT pathways may be linked to increased lung tumor growth (73). Impaired cholesterol efflux pathways due to downregulation of ABCA1 expression have also been shown to promote macular degeneration in mice (74). Finally, aberrations in HDL function have also been found in Fragile X, a genetic condition with a wide range of developmental disorders (76). Using iPSC generated human astrocytes, Talvio *et al*. found a dramatic decrease in ABCA1 expression in Fragile X patients, leading to the build-up of cholesterol (75).

In conclusion, while systemic inflammation generally leads to defective RCT and hence predictive of pre-clinical atherosclerosis, measurements of RCT may also be important for human conditions outside of cardiovascular disease.

## 4 RCT/CEC in response to therapeutic intervention

The number of human studies reporting changes in CEC upon therapeutic intervention remains relatively small and mostly been described in inflammatory states. We mostly discuss here changes in CEC upon anti-inflammatory treatments of two autoimmune diseases, namely psoriasis and SLE, but the effect of a therapeutic intervention on CEC has been examined for several other diseases and conditions including Familial Hypercholesterolemia and Rheumatoid Arthritis (77–81).

### 4.1 Psoriasis (PSO)

To date, five studies have investigated the role of different psoriasis treatments on CEC. While each treatment has proven successful in resolving skin inflammation, the effect on CEC has proven modest and inconsistent across therapies. The first reported study that measured CEC after PSO treatment was performed using the oral Janus kinase inhibitor, tofacitinib, where a difference in CEC between baseline and the 16-week end of study was not observed (82). A few months later, a randomized, double-blind trial which included an arm of PSO treatment with the TNFα blocking antibody adalimumab revealed a small improvement in CEC after 12 weeks, but it later decreased at the 52-week timepoint (83). Other potent anti-PSO treatments, such as secukinumab, which neutralizes the cytokine IL-17A, and ustekinumab, which blocks cytokines IL- 12 and IL-23, failed to demonstrate any significant improvement in CEC at any timepoint (84, 85). As an alternative to cytokine inhibition, the phosphodiesterase 4 inhibitor apremilast, is also used as a PSO treatment and is associated with weight loss (86). In a phase 4, open-label nonrandomized clinical trial Gelfand et al. demonstrated that although apremilast is generally beneficial to cardiometabolism, an improvement in CEC was not observed (86).

### 4.2 Systemic lupus erthematosus (SLE)

Therapeutic interventions for SLE include Peroxisome Proliferator-Activated Receptor (PPAR)-γ agonism (pioglitazone) and type 1 Interferon receptor (anifrolumab) or B cell-activating factor (BAFF) blockade. SLE patients undergoing treatment with these three therapies have been examined for changes to CEC (87–89). Blockade of the IFN receptor pathway for up to one year failed to improve CEC. Similarly, a small but not significant improvement in CEC was observed with pioglitazone treatment at the end of a 3- month study (87, 89). However, a recent study reported that a 6-month treatment of SLE patients with the BAFF blocking antibody belimumab, which inhibits B-cell activation and maturation, caused a significant improvement in CEC in SLE patients (88).

While the overall effect of anti-inflammatory therapeutics on CEC seem to be disappointing, it is plausible that optimal treatment time and the most potent CEC boosting pathways are yet to be determined. It would also be beneficial to perform studies with larger patient cohorts, which could be facilitated with one or more of the newer, higher throughput assays described below.

## 5 Cell-free RCT assays

Recently, several cell-free assays that closely correlate to the classic CEC assays done with cells for measuring HDL function have been developed, which vary in their potential for translation into a routine diagnostic test (90). Several cell-free HDL functionality assays measure CEC (91) or cholesterol uptake capacity (CUC) (92–94) by monitoring the efflux of fluorescent analogs of cholesterol from a lipid-coated substrate to apoB-depleted serum (Figures 1B,C). Recently, a fully automated immunoassay for CUC to assess HDL function and CVD risk has been developed (95). The latter assay appears to be promising, however, it relies on a monoclonal apoA-I antibody, which potentially introduces a selection bias as it does not capture non-apoA-I-containing HDL particles that also likely modulate CUC. Nevertheless, the rapid, high-throughput fully-automated CUC immunoassay, which highly correlates with CEC [81], appears to be a potential suitable surrogate assay for CEC.

Other means of HDL functionality assessment involve measurement of the rate of exogenous apoA-I exchange onto serum HDL by electron paramagnetic resonance (96, 97) or by using NBD/Alexa647 double-labeled ApoA-I, whose NBD/Alexa647 emission ratio increased upon exchange into HDL (98, 99) (Figures 1D,E). Several exchangeable plasma HDL apolipoproteins have been shown, however, to mediate both cellular cholesterol and phospholipid efflux (100). Since the aforementioned ApoA-I exchange assays use only exogenous ApoA-I as a probe, they are not able to fully assess endogenous exchangeable HDL apolipoprotein functionality.

We have developed a relatively simple and sensitive cell-free, HDL-specific phospholipid efflux (HDL-SPE) assay that is based on active solubilization of a non-exchangeable, head group-tagged fluorescent phosphatidylethanolamine (PE) from donor particles by endogenous exchangeable plasma HDL-derived apolipoproteins (ApoA-I, ApoA-II, ApoA-IV and ApoC-III) [90]. Whole sample plasma or serum and a pooled human reference plasma, are incubated with the fluoresent PE donor particles in 96-well plates for I hr at 37 ^o^ C with shaking (Figure 1F). The plates are centrifuged to pellet the dense donor lipid particles and supernatant HDL-associated PE fluorescence is measured and normalized to the reference plasma fluorescence [90]. HDL-SPE predicts incident CVD independent of traditional risk factors better than HDL-C and ApoA-I and is amenable for automation into a clinical diagnostic test (101).

The functionalities of HDL that underlies this assay includes the ability of the HDL-associated exchangeable lipoproteins to (i) dissociate from HDL particles (ii) bind to the surface of the donor lipid particle (iii) solubilize donor particle lipids and (iv) dissociate from the lipid particle along with the solubilized lipids. HDL-mediated phospholipid efflux is solely an active apolipoprotein-mediated process, as opposed to cholesterol-mediated efflux, which is based on equilibration of cholesterol pools on donor particle and acceptor lipoprotein surfaces via monomeric diffusion of cholesterol. Efflux of a non-transferable phospholipid on the other hand is HDL-specific and thus can be measured in whole plasma, whereas cholesterol efflux is non-specific and requires the use of ApoB-depleted plasma/serum (101, 102).

The multiple mechanistic steps underlying the HDL-SPE assay may be the reason why it was more strongly inversely associated with CAD in a CVD cohort than the cell-based CEC assay and was also more significantly inversely correlated with non-calcified plaque burden, total plaque burden, fibro-fatty burden and fibrous burden in CAD subjects assessed by coronary CT angiography (101). These findings suggest that HDL-SPE may be able assess the ability of HDL to solubilize excess extracellular lipid present in advanced arterial wall lesions, which tends to be more abundant than intracellular lipid in late atherosclerotic lesions (103). In this cohort, HDL-C was matched in CAD and non-CAD subjects. It is important to note that the cell-based CEC assay has been analyzed in other cohorts with plaque and has also been linked to non-calcified plaque.

## 6 HDL proteome and subspecies effect on CEC

The wide variety of subspecies and variability of composition of the HDL proteome has been shown consistently to play a major role in its functions (1). Some studies have shown that specific proteins such as apolipoprotein ApoA-I, ApoC-II, ApoC-III, and ApoA-IV differentially correlate with CEC. For instance, ApoA-I and ApoC-III are positively associated with high CEC, while ApoA-IV shows an inverse relationship in certain populations (104). Additionally, proteins like complement C3, ApoE, and plasminogen are inversely associated with CEC (104).

HDL subspecies also been shown to have an effect in cholesterol efflux. In particular, studies have shown that HDL3b and HDL3c are the most efficient mediators of cholesterol efflux via the ABCA1 pathway (105). These small HDL particles are enriched in negatively charged phospholipids, which enhance their functionality in cholesterol efflux and other activities (106). Conversely, large HDL particles are more effective in cholesterol efflux via the SR-BI pathway (107). Taken together, these observations still suggest that measuring the HDL proteome is not enough to completely explain the determinants in CEC in all HDL subspecies, but it does highlight the protein modifications and subspecies of lipids might be playing a role in CEC variations.

## 7 Concluding thoughts

Due to the now many clinical trials that have failed to show any benefit from raising HDL-C in lowering CVD risk, many investigators in the field are losing interest or even abandoning their research efforts in this area. Recent studies have provided convincing evidence for the existence of dysfunctional HDL (90, 101, 108), which has shifted interest from measuring HDL-C levels to measuring HDL functionality as a possible future metric of CVD risk. We have shown that the HDL-SPE assay, which can be readily translated into a relatively rapid automated diagnostic assay, is at least as good as CEC in assessing incident CVD risk. Other cell-free assays of HDL function, particularly the CUC [84] and exogenous apoA-I exchange rate [88] assays, also show great promise for being developed into new diagnostic tests. These cell-free assays apparently reflect complementary risk information distinct from cell-based CEC and may offer improved risk prediction.

There is presently a great need for a well standardized and fully automated assays to conduct large studies on the effects of new drugs on HDL function. It will also be important to perform baseline and follow-up measurements of HDL function after any type of therapeutic intervention. Ideally these types of studies on HDL function should be done throughout the course of a clinical trial and not just at baseline as has been done for most previous studies. Longer follow-up times are also needed before definitive conclusions can be made concerning their effectiveness.

Lipid-rich plaque with longer lipid length, wider lipid arc, and higher degree of stenosis has identified in patients at higher risk of future cardiac events (109). Insofar as HDL-SPE was significantly inversely correlated with noncalcified plaque and fibro-fatty plaque burden, it may also serve as an additional metric to assess the need for invasive cardiologic procedures, such as cardiac catheterization. Although the association of other cell-free assays with non-calcified plaque burden have yet to be determined, they may also prove to be useful for assessing CVD risk and thus aid cardiologists in the future.

In summary, the new cell-free HDL functionality assays, will likely quicken the pace of research for better understanding the mechanisms underlying HDL functionality, as well as factors that modulate lipid removal from arterial wall plaque. Such information may prove vital for the possible future development of HDL based drugs.
